# Efficient regio- and stereoselective access to novel fluorinated β-aminocyclohexanecarboxylates

**DOI:** 10.3762/bjoc.9.130

**Published:** 2013-06-17

**Authors:** Loránd Kiss, Melinda Nonn, Reijo Sillanpää, Santos Fustero, Ferenc Fülöp

**Affiliations:** 1 University of Szeged, Institute of Pharmaceutical Chemistry, Szeged, Hungary; 2 Stereochemistry Research Group of the Hungarian Academy of Sciences, University of Szeged, H-6720 Szeged, Eötvös 6, Hungary; 3 Department of Chemistry, University of Jyväskylä, FIN-40014, Jyväskylä, Finland; 4 Universidad de Valencia, Facultad de Farmàcia, Departamento de Química Orgánica, Valencia, Spain

**Keywords:** amino acids, epoxidation, fluorination, hydroxylation, stereoselective reaction

## Abstract

A regio- and stereoselective method has been developed for the synthesis of novel fluorinated 2-aminocyclohexanecarboxylic acid derivatives with the fluorine attached to position 4 of the ring. The synthesis starts from either *cis*- or *trans*-β-aminocyclohex-4-enecarboxylic acids and involves regio- and stereoselective transformation of the ring C–C double bond through iodooxazine formation and hydroxylation, followed by hydroxy–fluorine or oxo–fluorine exchange.

## Introduction

Fluorine chemistry is an expanding area of research that has generated increasing interest in pharmaceutical and medicinal chemistry in recent years because of its considerable impact in drug discovery. There is currently extensive research activity in synthetic chemistry for the preparation of various biologically active fluorinated products [1–10].

Of special interest among such materials are the fluorinated amino acids, which in most cases exhibit higher bioactivities than the nonfluorinated counterparts. The fluorinated α- or acyclic β-amino acids have acquired significance as antibacterial or antifungal agents, enzyme inhibitors or as antitumoral compounds. Introduction of a fluorinated amino acid into a peptide may generate specific protein–ligand or protein–protein interactions, thereby determining thermal or metabolic stabilities, which is of great importance in peptide-based drug research [11–35]. These changes in properties may be more appreciable in the case of peptide oligomers formed from conformationally restricted fluorinated amino acids. Although cyclic β-amino acids are of major interest in pharmaceutical chemistry and in peptide research [36–60], only a relatively small number of fluorinated derivatives of this class of compounds have been synthesized so far [61–70].

## Results and Discussion

We recently developed a synthetic method for the regio- and stereoselective introduction of a fluorine atom onto the skeleton of a β-aminocyclohexanecarboxylic acid. The synthesis starts from the Boc-protected 2-aminocyclohex-4-enecarboxylic acid or 2-aminocyclohex-3-enecarboxylic acid and involves ring C–C bond transformation by regio- and stereoselective hydroxylation via iodolactonization, followed by hydroxy–fluorine exchange. This protocol was applied to synthesize fluorinated β-aminocyclohexane scaffolds with the fluorine atom on either position 3 or 5 of the ring. Whereas the procedure is a convenient economical route to fluorinated cyclohexane or cyclohexene β-amino acids, it did not allow extension to the synthesis of similar derivatives with the fluorine atom on position 4.

During our work performed to fill this gap, we have developed a synthetic procedure for gaining access to fluorinated β-aminocyclohexanecarboxylic acids.

**Scheme 1:**
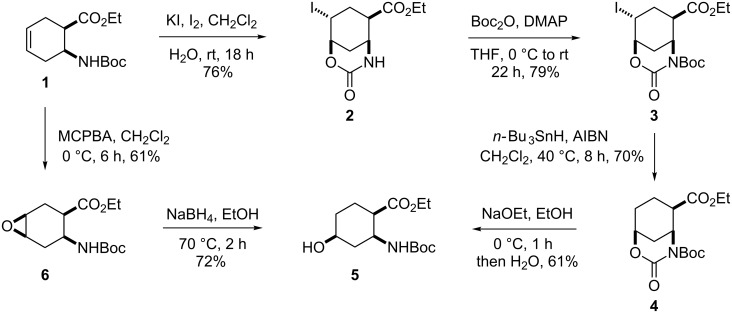
Synthesis of all-*cis* ethyl 4-hydroxylated β-aminocyclohexanecarboxylate **5**.

**Figure 1:**
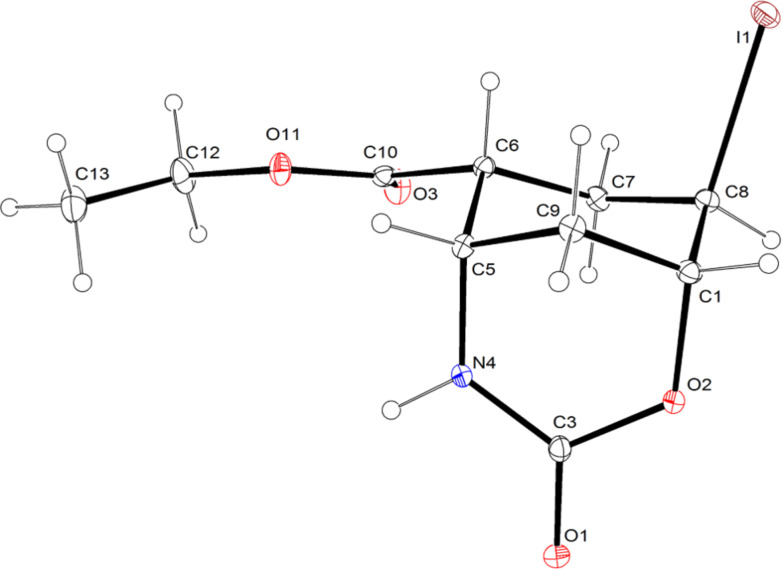
ORTEP diagram of iodooxazinone **2.** Thermal ellipsoids have been drawn at the 20 % probability level.

**Figure 2:**
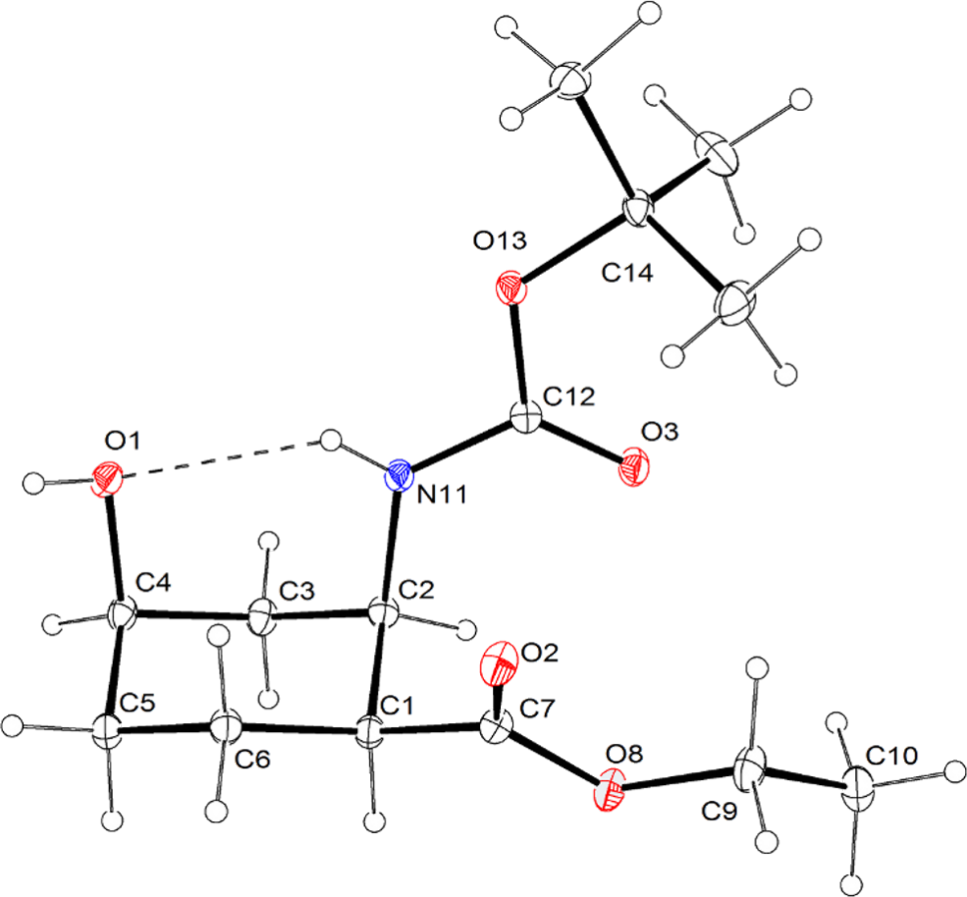
ORTEP diagram of hydroxylated amino ester **5**. Thermal ellipsoids have been drawn at the 20% probability level.

This synthesis starts from ethyl *cis*-2-aminocyclohex-4-enecarboxylate **1** [57] and follows two different strategies. One is based on regio- and stereoselective hydroxylation via iodooxazine formation, followed by fluorination, while the other includes stereoselective epoxidation and regioselective oxirane opening, followed by hydroxy–fluorine exchange. In the former protocol, amino ester **1** is treated with KI/I_2_ in H_2_O/CH_2_Cl_2_, which affords iodooxazinone derivative **2** stereo- and regioselectively (Scheme 1, Figure 1). Next, compound **2** is transformed to **3** by amide *N*-Boc protection with Boc_2_O and 4-dimethylaminopyridine (DMAP) in THF. Removal of the iodine from the cyclohexane skeleton in **3** is accomplished under reductive conditions. On treatment with *n*-Bu_3_SnH in the presence of a catalytic amount of azobisisobutyronitrile (AIBN) in dichloromethane under reflux, **3** undergoes deiodination to give ester **4** in 70% yield. Oxazinone **4** is then subjected to heterocycle ring opening with NaOEt in EtOH at 0 °C to furnish all*-cis* hydroxylated amino ester **5** with the hydroxy group on position 4 of the skeleton (Scheme 1, Figure 2).

Hydroxylated amino ester **5** was also prepared via the alternative route involving stereoselective epoxidation. Cyclohexene β-amino ester **1** underwent C–C double bond oxidation with 3-chloroperbenzoic acid (MCPBA) to afford epoxy amino ester **6** *cis*-diastereoselectively [57] (Scheme 1). Opening of the oxirane ring in **6** with NaBH_4_ in EtOH at 70 °C proceeded regioselectively, providing exclusively amino ester **5** with the hydroxy function on position 4 (for analogous transformations, see reference [60]).

**Scheme 2:**
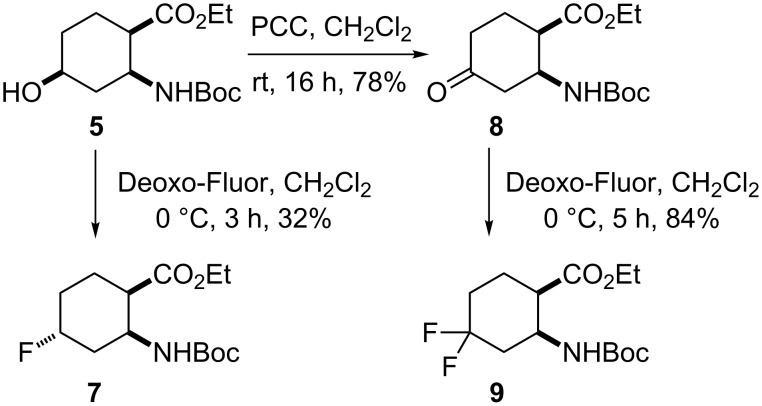
Syntheses of fluorinated amino esters **7** and **9**.

Hydroxylated amino ester **5** was next further used as a key compound for the synthesis of fluorinated target materials. A fluorine atom was introduced by hydroxy–fluorine exchange with bis(dimethoxyethylaminosulfur trifluoride) (Deoxo-Fluor®) reagent. The reaction was carried out under different experimental conditions, with variation of the temperature (−40 °C, 0 °C or 20 °C) and the solvent (toluene, CH_2_Cl_2_ or THF). Finally, it was found that hydroxylated amino ester **5** underwent inversion on reaction with a 50% Deoxo-Fluor toluene solution in CH_2_Cl_2_ at 0 °C [68–69] to give monofluorinated cyclohexane amino ester **7** in 32% yield (Scheme 2). This rather modest yield is attributed to the relatively large amount of elimination materials (40% overall). In continuation, the geminal difluorinated β-aminocyclohexanecarboxylic acid derivative with the fluorine atoms on position 4 was efficiently synthesized. Oxidation of the hydroxy group of amino ester **5** with pyridinium chlorochromate (PCC) in CH_2_Cl_2_ yielded the corresponding oxo-group-containing amino ester **8**, which was then converted with Deoxo-Fluor in CH_2_Cl_2_ at 0 °C to the corresponding geminal difluoro amino ester **9** in good yield (Scheme 2).

**Scheme 3:**
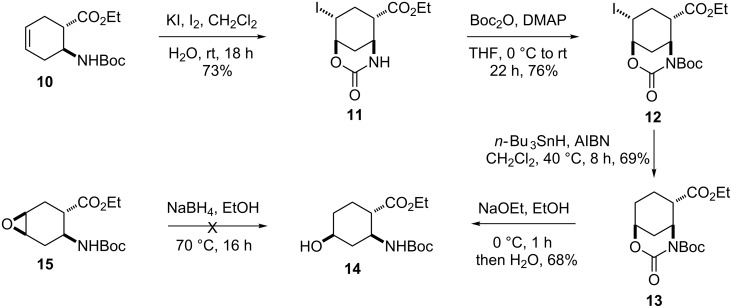
Synthesis of ethyl 4-hydroxy-β-aminocyclohexanecarboxylate **14**.

**Figure 3:**
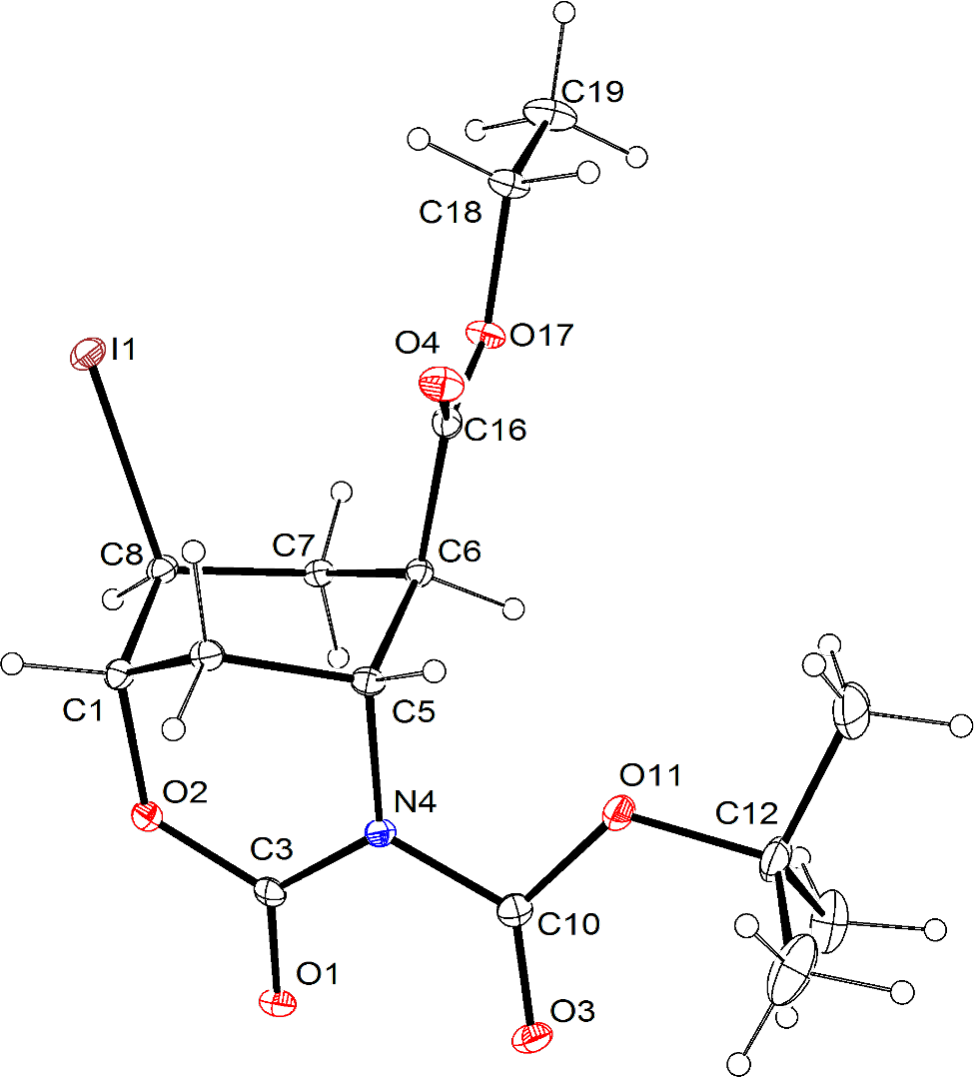
ORTEP diagram of iodooxazinone derivative **12**. Thermal ellipsoids have been drawn at the 20% probability level.

**Figure 4:**
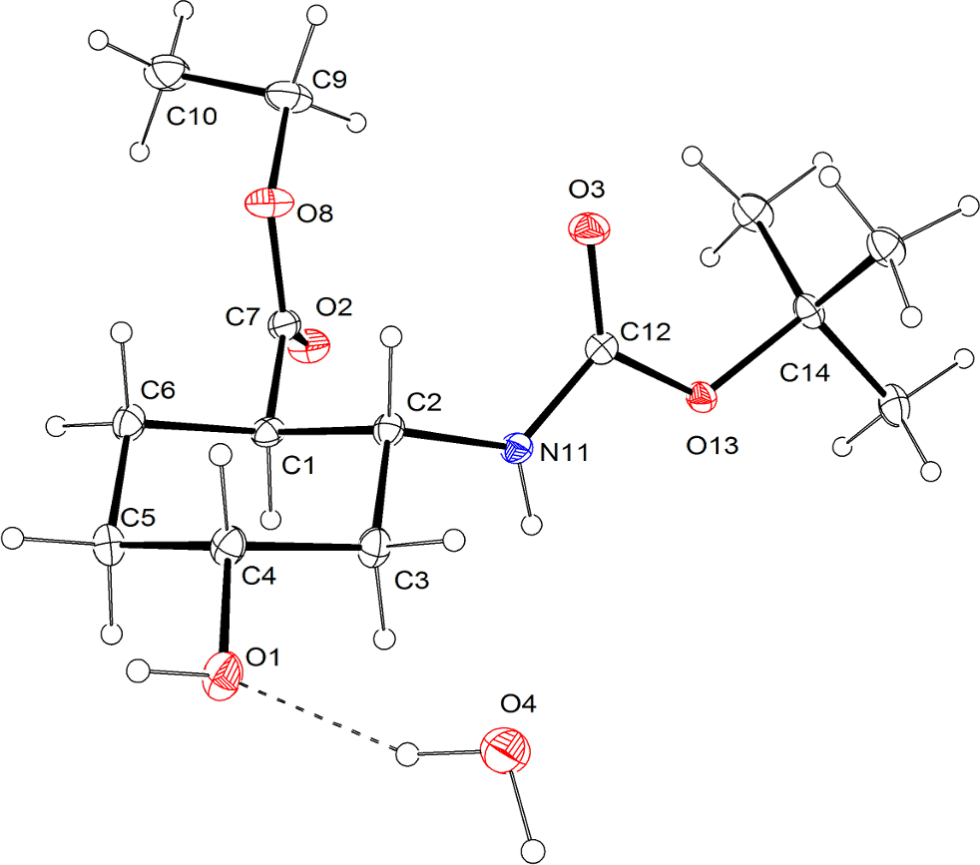
ORTEP diagram of hydroxylated amino ester **14**. The water molecule oxygen atom O4 is situated on the twofold axis with a population parameter of 0.6. Thermal ellipsoids have been drawn at the 20% probability level.

The synthetic route presented above could be extended to the preparation of other 4-fluorinated cyclohexane amino acid derivatives, stereoisomers of **7** or **9**. Ethyl *trans*-2-aminocyclohex-4-enecarboxylate **10** [57] was analogously transformed to its *cis* counterpart through regio- and stereoselective iodooxazine formation with KI/I_2_ to give compound **11** (Scheme 3). *N*-Protection of **11**, followed by reductive deiodination, proceeded via **12** (Figure 3) to afford ester **13**. Opening of the heterocyclic ring with NaOEt in EtOH at 0 °C furnished 4-hydroxylated amino ester **14**, a stereoisomer of **5** (Scheme 3, Figure 4).

It is noteworthy that in this latter case hydroxylated amino ester **14** could not be prepared by the alternative diastereoselective epoxidation and regioselective oxirane opening strategy: according to our previous results, the opening of epoxide **15** derived from **10** proceeded via a *trans*-diaxial chair conformation with the nucleophile attack on C4, thereby providing the 5-hydroxylated derivative [57,60].

**Scheme 4:**
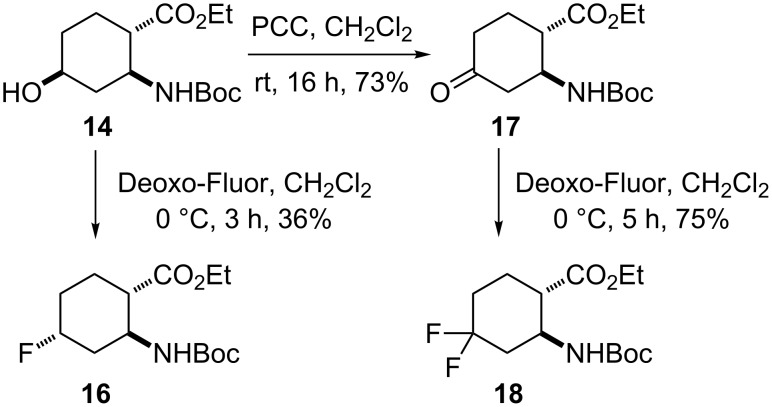
Syntheses of fluorinated amino esters **16** and **18**.

Next, hydroxylated cyclohexane amino ester **14** was converted with Deoxo-Fluor in CH_2_Cl_2_ at 0 °C to 4-fluorinated ethyl β-aminocyclohexanecarboxylate **16**, a stereoisomer of **7**. Unfortunately, the formation of a substantial amount of a mixture of elimination material could again not be avoided. When subjected to oxidation with PCC, amino ester **14** gave the corresponding oxo ester **17**, treatment of which with Deoxo-Fluor in CH_2_Cl_2_ at 0 °C provided geminal 4,4-difluorinated cyclohexane amino ester **18**, a stereoisomer of **9** (Scheme 4).

## Conclusion

In conclusion, a simple and convenient procedure has been developed for the introduction of one or two fluorine atoms onto the skeleton of either *cis*- or *trans*-β-aminocyclohexanecarboxylates. The synthetic concept involves regio- and stereoselective hydroxylation via iodooxazine formation, followed by hydroxy–fluorine or oxo–fluorine exchange.

## Supporting Information

File 1 Experimental procedures and characterization of compounds.
