# Supplementary material for: Efficient regio- and stereoselective access to novel fluorinated β-aminocyclohexanecarboxylates
Source: Beilstein J Org Chem. 2013 Jun 17;9:1164–9. doi: 10.3762/bjoc.9.130 (PMC3701410; doi:10.3762/bjoc.9.130)
Supplement: File 1 — Experimental procedures and characterization of compounds. [file Beilstein_J_Org_Chem-09-1164-s001.pdf]

Supporting Information

for

Efficient regio- and stereoselective access to novel fluorinated β-aminocyclohexanecarboxylates

Loránd Kiss[1], Melinda Nonn[2], Reijo Sillanpää[3], Santos Fustero[4] and Ferenc Fülöp[1,2*]

Address: [1]University of Szeged, Institute of Pharmaceutical Chemistry, Szeged, Hungary, [2]Stereochemistry Research Group of the Hungarian Academy of Sciences, University of Szeged, H-6720 Szeged, Eötvös 6, Hungary, [3]Department of Chemistry, University of Jyväskylä, FIN-40014, Jyväskylä, Finland and [4]Universidad de Valencia, Facultad de Farmàcia, Departamento de Química Orgánica, Valencia, Spain

Email: Ferenc Fülöp - fulop@pharm.u-szeged.hu

*Corresponding author

Experimental procedures and characterization of compounds

Experimental

The chemicals were purchased from Sigma–Aldrich. The NMR spectra were recorded at 400 MHz in $CDCl_3$ or DMSO as solvent, with tetramethylsilane as the internal standard. The solvents were used as received from the suppliers. Melting points were determined with a Kofler apparatus. Elemental analyses were recorded on a Perkin-Elmer CHNS-2400 Ser II Elemental Analyzer. Silica gel 60 F254 was purchased from Merck.

General procedure for iodooxazine formation

To a solution of β-amino ester (**1** or **10**, 2.68 g, 10 mmol) in CH_2Cl_2 (60 mL), H_2O (60 mL), KI (1.5 equiv) and I_2 (1.5 equiv) were added and the mixture was stirred at room temperature for 18 h. It was then diluted with CH_2Cl_2 (60 mL) and washed with saturated aqueous Na_2SO_3 solution (3 x 50 mL). The organic layer was next dried over Na_2SO_4 and concentrated under reduced pressure to give **2** or **11** as a yellowish, white solid, which was crystallized from Et_2O.

Ethyl (1*R**,5*S**,6*R**,8*R**)-8-iodo-3-oxo-2-oxa-4-azabicyclo[3.3.1]nonane-6-carboxylate (2)

A yellowish-white solid (Et_2O); mp 154–156 °C; yield: 69% (R_f 0.25, *n*-hexane/EtOAc 1:2); ^{1}H NMR (400 MHz, $CDCl_3$) δ 1.20 (t, 3H, *t*-Bu), 1.88-2.07 (m, 3H, CH_2), 2.50-2.58 (m, 1H, CH_2), 2.78-2.85 (m, 1H, H-6), 3.69-3.74 (m, 1H, H-5), 4.02-4.12 (m, 2H, OCH_2), 4.55-4.60 (m, 1H, H-1), 4.71-4.75 (m, 1H, H-8), 7.63 (brs, 1H, N-H); ^{13}C NMR (100 MHz, DMSO) δ 20.5, 25.8, 28.6, 29.2, 43.6, 47.1, 61.4, 75.9, 153.1, 172.2; ESIMS (pos) *m*/*z*: 362.1 (M + Na); Anal. calcd for $C_{10}H_{14}INO_4$: C, 35.42; H, 4.16; N, 4.13; found: C, 35.63; H, 4.44; N, 3.87.

Ethyl (1*R,5*S**,6*S**,8*R**)-8-iodo-3-oxo-2-oxa-4-azabicyclo[3.3.1]nonane-6-carboxylate (11)**

A yellowish-white solid (Et_2O); mp 75–76 °C; yield: 71% (R_f 0.2, *n*-hexane/EtOAc 1:2); ^{1}H NMR (400 MHz, $CDCl_3$) δ 1.24 (t, 3H. CH_3), 1.41 (s, 9H, *t*-Bu), 2.01-2.09 (m, 1H, CH_2), 2.51-2.62 (m, 2H, CH_2), 2.76-2.80 (m, 1H, CH_2), 2.98-3.06 (m, 1H, H-6), 3.98-4.03 (m, 1H, H-8), 4.18-4.26 (m, 2H, OCH_2), 4.56-4.60 (m, 1H, H-1), 4.78-4.81 (m, 1H, H-5), 6.49 (brs, 1H, N-H); ^{13}C NMR (100 MHz, DMSO) δ 14.8, 22.4, 25.9, 27.8, 43.1, 41.1, 61.4, 76.7, 153.2, 172.4; ESIMS (pos) *m*/*z*: 362.0 (M + Na); Anal. calcd for $C_{10}H_{14}INO_4$: C, 35.42; H, 4.16; N, 4.13; found: C, 35.76; H, 4.40; N, 3.85.

General procedure for the *N*-Boc protection of oxazinones 2 and 11

To a solution of oxazinone (**2** or **11**, 3.39 g, 10 mmol) in THF (70 mL), Boc_2O (1 equiv), DMAP (300 mg) and Et_3N (1.5 equiv) were added at 0 °C and the mixture was further stirred at room temperature for 22 h. It was then diluted with EtOAc (110 mL), washed with water (2 x 60 mL), dried over Na_2SO_4 and concentrated under reduced pressure. The crude material was purified by means of column chromatography on silica gel (*n*-hexane/EtOAc) to give **3** or **12** as a white solid.

4-*tert*-Butyl-6-ethyl (1*R,5*S**,6*R**,8*R**)-8-iodo-3-oxo-2-oxa-4-azabicyclo[3.3.1]nonane-4,6-dicarboxylate (3)**

A white solid; mp 126–128 °C; yield: 69% (R_f 0.6, *n*-hexane/EtOAc 3:1); ^{1}H NMR (400 MHz, $CDCl_3$) δ 1.26 (t, 3H, CH_3), 1.45 (s, 9H, *t*-Bu), 2.03-2.11 (m, 2H, CH_2), 2.32-2.39 (m, 1H, CH_2), 2.74-2.81 (m, 1H, CH_2), 2.89-2.96 (m, 1H, H-6), 4.02-4.10 (m, 2H, OCH_2), 4.58-4.61 (m, 1H, H-5), 4.65-4.66 (m, 1H, H-1), 4.89-4.91 (m, 1H, H-8); ^{13}C NMR (100 MHz, DMSO) δ 14.7, 26.2, 26.7, 28.4, 28.9, 42.7, 50.9, 61.4, 77.1, 84.1, 148.3, 152.5,

172.1; ESIMS (pos) *m*/*z*: 462.04 (M + Na); Anal. calcd for $C_{15}H_{22}INO_6$: C, 41.02; H, 5.05; N, 3.19; found: C, 41.35; H, 4.84; N, 3.41.

4-*tert*-Butyl-6-ethyl (1*R,5*S**,6*S**,8*R**)-8-iodo-3-oxo-2-oxa-4-azabicyclo[3.3.1]nonane-4,6-dicarboxylate (12)**

A white solid (Et_2O); mp 109–111 °C; yield: 76% (R_f 0.5, *n*-hexane/EtOAc 3:1); ^1H NMR (400 MHz, $CDCl_3$) δ 1.26 (t, 3H, CH_3), 1.55 (s, 9H, *t*-Bu), 2.09-2.18 (m, 1H, CH_2), 2.43-2.50 (m, 1H, CH_2), 2.61-2.68 (m, 1H, CH_2), 2.88-2.94 (m, 1H, CH_2), 3.08-3.17 (m, 1H, H-6), 4.18-4.27 (m, 2H, OCH_2), 4.49-4.53 (m, 1H, H-8), 4.74-4.78 (m, 1H, H-5), 4.79-4.73 (m, 1H, H-1); ^{13}C NMR (100 MHz, DMSO) δ 14.8, 22.9, 24.3, 27.5, 28.4, 41.1, 50.1, 61.6, 77.6, 84.0, 152.5, 172.2, 208.0; ESIMS (pos) *m*/*z*: 462.11 (M + Na); Anal. calcd for $C_{15}H_{22}INO_6$: C, 41.02; H, 5.05; N, 3.19; found: C, 40.83; H, 4.80; N, 3.43.

General procedure for the deiodination reaction

To a solution of iodooxazinone **3** or **12** (2.2 g, 5 mmol) in dry CH_2Cl_2 (60 mL), *n*-Bu_3SnH (2 equiv) and AIBN (400 mg) were added and the mixture was stirred under reflux for 8 h. The solvent was then evaporated under reduced pressure and the crude product was purified by column chromatography on silica gel (*n*-hexane/EtOAc).

4-*tert*-Butyl-6-ethyl (1*S,5*S**,6*R**)-3-oxo-2-oxa-4-azabicyclo[3.3.1]nonane-4,6-dicarboxylate (4)**

A white solid (Et_2O); mp 112–114 °C; yield: 72% (R_f 0.5, *n*-hexane/EtOAc 2:1); ^1H NMR (400 MHz, $CDCl_3$) δ 1.24 (t, 3H, CH_3), 1.51 (s, 9H, *t*-Bu), 1.52-1.62 (m, 1H, CH_2), 1.77-1.83 (m, 1H, CH_2), 1.90-2.11 (m, 2H, CH_2), 2.18-2.24 (m, 2H, CH_2), 2.57-2.61 (m, 1H, H-6), 4.04-4.12 (m, 2H, OCH_2), 4.64-4.68 (m, 1H, H-5), 4.98-5.02 (m, 1H, H-1); ^{13}C NMR (100 MHz,

DMSO) δ 14.7, 18.8, 28.8, 29.7, 29.9, 46.6, 51.3, 61.0, 74.3, 83.5, 149.3, 152.9, 172.9; ESIMS (pos) *m/z*: 336.18 (M + Na); Anal. calcd for $C_{15}H_{23}NO_6$: C, 57.50; H, 7.40; N, 4.47; found: C, 57.22; H, 7.12; N, 4.69.

4-*tert*-Butyl-6-ethyl (1*S,5*S**,6*S**)-3-oxo-2-oxa-4-azabicyclo[3.3.1]nonane-4,6-dicarboxylate (13)**

A yellow oil; yield: 69% (R_f 0.45, *n*-hexane/EtOAc 2:1); ^{1}H NMR (400 MHz, DMSO) δ 1.20 (t, 3H, CH_3), 1.43 (s, 9H, *t*-Bu), 1.58-2.10 (m, 6H, CH_2), 2.91-2.99 (m, 1H, H-6), 4.11-4.19 (m, 2H, OCH_2), 4.61-4.68 (m, 2H, H-1 and H-4); ^{13}C NMR (100 MHz, DMSO) δ 14.8, 14.9, 26.5, 26.7, 28.3, 43.3, 51.8, 61.4, 74.4, 83.6, 149.2, 151.4, 172.2; ESIMS (pos) *m/z*: 336.27 (M + Na); Anal. calcd for $C_{15}H_{23}NO_6$: C, 57.50; H, 7.40; N, 4.47; found: C, 57.19; H, 7.16; N, 4.12.

General procedure for the preparation of hydroxylated amino esters

Synthesis from oxazinones:

To a solution of oxazinone **4** or **13** (1.13 g, 5 mmol) in EtOH (20 mL), NaOEt (1 equiv) was added at 0 °C and the mixture was stirred at this temperature for 1 h. Next, water (20 mL) was added to the mixture and it was extracted with CH_2Cl_2 (3 x 20 mL). The combined organic layers were dried (Na_2SO_4) and concentrated under reduced pressure. The crude product was purified by means of column chromatography on silica gel (*n*-hexane/EtOAc).

Synthesis from epoxide:

To a solution of epoxyamino ester **6** [1] (855 mg, 3 mmole) in EtOH (25 mL), $NaBH_4$ (2 equiv) was added and the mixture was stirred at 70 °C for 2 h. It was then diluted with H_2O (30 mL) and extracted with CH_2Cl_2 (3 x 20 mL), the combined organic layers were dried

(Na_2SO_4) and the solvent was evaporated off under reduced pressure. The crude product was purified by column chromatography on silica gel (*n*-hexane/EtOAc), giving compound **5**.

Ethyl (1*R,2*S**,4*S**)-2-(*tert*-butoxycarbonylamino)-4-hydroxycyclohexanecarboxylate (5)**

A white solid; mp 64–65 °C; yield: 68% (66%) (R_f 0.4, *n*-hexane/EtOAc 1:1); ^{1}H NMR (400 MHz, DMSO) δ 1.19 (t, 3H, CH_3, *J* = 7.20 Hz), 1.39 (s, 9H, *t*-Bu), 1.40-1.48 (m, 1H, CH_2), 1.53-1.59 (m, 2H, CH_2), 1.66-1.75 (m, 2H, CH_2), 1.84-1.93 (m, 1H, CH_2), 2.63-2.68 (m, 1H, H-1), 3.67-3.73 (m, 1H, H-2), 3.79-3.85 (m, 1H, H-4), 3.97-4.04 (m, 2H, OCH_2), 4.83 (brs, 1H, O-H), 6.39 (brs, 1H, N-H); ^{13}C NMR (100 MHz, DMSO) δ 14.8, 21.1, 30.9, 31.6, 37.3, 44.5, 49.0, 60.5, 66.8, 78.6, 155.2, 173.7; ESIMS (pos) *m*/*z*: 310.27 (M + Na); Anal. calcd for $C_{14}H_{25}NO_5$: C, 58.52; H, 8.77; N, 4.87; found: C, 58.24; H, 8.99; N, 4.52.

Ethyl (1*S,2*S**,4*S**)-2-(*tert*-butoxycarbonylamino)-4-hydroxycyclohexanecarboxylate (14)**

A white solid; mp 114–116 °C; yield: 73% (R_f 0.35, *n*-hexane/EtOAc 1:1); ^{1}H NMR (400 MHz, DMSO) δ 1.01-1.12 (m, 1H, CH_2), 1.18 (t, 3H, CH_3, *J* = 7.15 Hz), 1.38 (s, 9H, *t*-Bu), 1.38-1.42 (m, 2H, CH_2), 1.76-1.82 (m, 2H, CH_2), 1.89-1.93 (m, 1H, CH_2), 2.17-2.23 (m, 1H, H-1), 3.38-3.43 (m, 1H, H-2), 3.43-3.48 (m, 1H, H-4), 3.96-4.09 (m, 2H, OCH_2), 4.62 (brs, 1H, O-H), 6.81 (brs, 1H, N-H); ^{13}C NMR (100 MHz, DMSO) δ 14.9, 26.3, 29.0, 34.3, 40.2, 49.0, 50.2, 60.5, 67.9, 78.3, 155.4, 174.5; ESIMS (pos) *m*/*z*: 310.36 (M + Na); Anal. calcd for $C_{14}H_{25}NO_5$: C, 58.52; H, 8.77; N, 4.87; found: C, 58.20; H, 8.46; N, 4.55.

General procedure for the oxidation of hydroxylated amino esters

To a solution of hydroxylated amino ester **5** or **14** (287 mg, 1 mmol) in CH_2Cl_2 (15 mL), PCC (1.3 equiv) was added and the mixture was stirred at room temperature for 16 h. It was then diluted with CH_2Cl_2 (20 mL), washed with water (2 x 15 mL), dried over Na_2SO_4, and

concentrated under reduced pressure. The crude product was purified by means of column chromatography on silica gel (*n*-hexane/EtOAc).

Ethyl (1*R,2*S**)-2-*tert*-butoxycarbonylamino-4-oxocyclohexanecarboxylate (8)**

A white solid; mp 72–74 °C; yield: 78% (R_f 0.50, *n*-hexane/EtOAc 1:1); ^{1}H NMR (400 MHz, DMSO) δ 1.23 (t, 3H, CH_3, *J* = 7.10 Hz), 1.42 (s, 9H, *t*-Bu), 1.78-1.83 (m, 1H, CH_2), 2.18-2.26 (m, 4H, CH_2 and H-1), 2.60-2.66 (m, 1H, CH_2), 3.04-3.10 (m, 1H, CH_2), 3.97-4.09 (m, 2H, OCH_2), 4.42-4.48 (m, 1H, H-2), 7.02 (brs, 1H, N-H); ^{13}C NMR (100 MHz, DMSO) δ 14.8, 22.8, 29.0, 30.0, 39.8, 43.8, 46.8, 50.4, 60.8, 78.7, 158.0, 172.9, 208.7; ESIMS (pos) *m*/*z*: 308.63 (M + Na); Anal. calcd for $C_{14}H_{23}NO_5$: C, 58.93; H, 8.12; N, 4.91; found: C, 58.65; H, 8.33; N, 4.68.

Ethyl (1*S,2*S**)-2-*tert*-butoxycarbonylamino-4-oxocyclohexanecarboxylate (17)**

A white solid; mp 79–82 °C; yield: 71%; (R_f 0.45, *n*-hexane/EtOAc 1:1); ^{1}H NMR (400 MHz, DMSO) δ 1.22 (t, 3H, CH_3, *J* = 7.10 Hz), 1.40 (s, 9H, *t*-Bu), 1.70-1.77 (m, 1H, CH_2), 2.02-2.08 (m, 1H, CH_2), 2.22-2.29 (m, 1H, CH_2), 2.33-2.48 (m, 3H, CH_2), 2.68-2.74 (m, 1H, H-1), 3.78-3.85 (m, 1H, H-2), 4.02-4.11 (m, 2H, OCH_2), 7.06 (brs, 1H, N-H); ^{13}C NMR (100 MHz, DMSO) δ 14.9, 25.8, 29.4, 39.4, 40.9, 41.1, 51.3, 60.9, 78.7, 155.3, 173.6, 208.1; ESIMS (pos) *m*/*z*: 308.56 (M + Na); Anal. calcd for $C_{14}H_{23}NO_5$: C, 58.93; H, 8.12; N, 4.91; found: C, 58.63; H, 7.84; N, 4.63.

General procedure for monofluorination

To a solution of hydroxyamino ester (287 mg, 1 mmol) in CH_2Cl_2 (10 mL) under an Ar atmosphere, 50% Deoxofluor in toluene (2 equiv) was added and the solution was stirred at 20 °C for 3 h. It was then diluted with CH_2Cl_2 (30 mL) and the organic layer was washed with

saturated aqueous $NaHCO_3$ solution (2 x 20 mL), dried (Na_2SO_4) and concentrated. The crude product was purified by column chromatography on silica gel (*n*-hexane/EtOAc 9:1).

Ethyl (1*R,2*S**,4*R**)-2-(*tert*-butoxycarbonylamino)-4-fluorocyclohexanecarboxylate (7)**

A white solid; mp 74–76 °C; yield: 32% (R_f 0.45, *n*-hexane/EtOAc 4:1); ^{1}H NMR (400 MHz, $CDCl_3$) δ 1.23 (t, 3H, CH_3, *J* = 7.15 Hz), 1.36 (s, 9H, *t*-Bu), 1.71-2.07 (m, 6H, CH_2), 2.78-2.84 (m, 1H, H-1), 4.02-4.14 (m, 3H, H-2 and OCH_2), 4.69-4.86 (m, 1H, H-4), 5.19 (brs, 1H, N-H); ^{13}C NMR (400 MHz, $CDCl_3$) δ 14.6, 22.5, 27.3, 28.8, 34.5, 44.1, 45.6, 60.9, 80.6, 88.1, 90.3, 155.4, 161.5; ^{19}F NMR (100 MHz, $CDCl_3$) δ −184.1; ESIMS (pos) *m*/*z*: 312.3 (M + Na). Anal. calcd for $C_{14}H_{24}FNO_4$: C, 58.11; H, 8.36; N, 4.84; found: C, 57.83; H, 8.05; N, 4.51.

Ethyl (1*S,2*S**,4*R**)-2-(*tert*-butoxycarbonylamino)-4-fluorocyclohexanecarboxylate (16)**

A white solid; mp 102–104 °C; yield: 36% (R_f 0.40, *n*-hexane/EtOAc 4:1); ^{1}H NMR (400 MHz, DMSO) δ 1.22 (t, 3H, CH_3, *J* = 7.15 Hz), 1.34 (s, 9H, *t*-Bu), 1.48-1.52 (m, 2H, CH_2), 1.70-2.02 (m, 3H, CH_2), 2.21-2.29 (m, 1H, CH_2), 2.30-2.48 (m, 1H, H-1), 3.79-3.93 (m, 1H, H-2), 4.03-4.16 (m, 2H, OCH_2), 4.50 (brs, 1H, N-H), 4.75-4.95 (m, 1H, H-4); ^{13}C NMR (100 MHz, DMSO) δ 14.6, 23.3, 28.7, 29.6, 36.9, 47.6, 49.1, 61.1, 79.8, 87.6, 90.0, 155.2, 173.9; ^{19}F NMR (400 MHz, $CDCl_3$) δ −184.4; ESIMS (pos) *m*/*z*: 312.27 (M + Na); Anal. calcd for $C_{14}H_{24}FNO_4$: C, 58.11; H, 8.36; N, 4.84; found: C, 57.81; H, 8.61; N, 4.52.

General procedure for difluorination

To a solution of ketoester (143 mg, 0.5 mmole) in CH_2Cl_2 (5 mL) under an Ar atmosphere, 50% Deoxofluor in toluene (4 equiv) was added and the solution was stirred at 20 °C for 5 h. It was then diluted with CH_2Cl_2 (20 mL) and the organic layer was washed with saturated

aqueous $NaHCO_3$ solution (2 x 20 mL), dried (Na_2SO_4) and concentrated. The crude product was purified by column chromatography on silica gel (*n*-hexane-EtOAc 9:1).

Ethyl (1*R,2*S**)-2-(*tert*-butoxycarbonylamino)-4,4-difluorocyclohexanecarboxylate (9)**

A white solid; mp 62–64 °C; yield: 84% (R_f 0.35, *n*-hexane/EtOAc 4:1); ^1H NMR (400 MHz, DMSO) δ 1.22 (t, 3H, CH_3, J = 7.15 Hz), 1.33 (s, 9H, *t*-Bu), 1.69-2.55 (m, 6H, CH_2, 2.68-2.74 (m, 1H, H-1), 4.02-4.13 (m, 3H, H-2 and OCH_2), 5.26 (brs, 1H, N-H); ^{13}C NMR (100 MHz, DMSO) δ 14.5, 20.9, 22.6, 28.7, 31.5, 37.9, 43.6, 61.2, 80,1, 123.0, 155.3, 173.7; ^{19}F NMR (400 MHz, $CDCl_3$) δ −91.3, −92.5; ESIMS (pos) *m*/*z*: 330.52 (M + Na); Anal. calcd for $C_{14}H_{23}F_2NO_4$: C, 54.71; H, 7.54; N, 4.56; found: C, 54.45; H, 7.22; N, 4.79.

Ethyl (1*S,2*S**)-2-(*tert*-butoxycarbonylamino)-4,4-difluorocyclohexanecarboxylate (18)**

A white solid; mp 113–115 °C; yield: 75% (R_f 0.30, *n*-hexane/EtOAc 4:1); ^1H NMR (400 MHz, DMSO) δ 1.23 (t, 3H, CH_3, J = 7.15 Hz), 1.30 (s, 9H, *t*-Bu), 1.58-2.08 (m, 5H, CH_2), 2.26-2.41 (m, 1H, CH_2), 2.48-2.55 (m, 1H, H-1), 3.94-4.02 (m, 1H, H-2), 4.05-4.13 (m, 2H, OCH_2), 4.73 (brs, 1H, N-H); ^{13}C NMR (100 MHz, DMSO) δ 14.6, 23.0, 28.7, 32.2, 38.6, 46.7, 48.7, 61.4, 80.2, 122.0, 155.0, 173.1; ^{19}F NMR (400 MHz, $CDCl_3$) δ −91.5, 92.4; ESIMS (pos) *m*/*z*: 330.50 (M + Na); Anal. calcd for $C_{14}H_{23}F_2NO_4$: C, 54.71; H, 7.54; N, 4.56; found: C, 54.48; H, 7.28; N, 4.81.

Crystallography: Crystallographic data for compounds **2**, **12** and **14** were collected with a Nonius-Kappa diffractometer equipped with a CCD area-detector using Mo Kα radiation (λ = 0.71073 Å), and for compound **5** with an Agilent Supernova diffractometer equipped with an Atlas area-detector, using Cu Kα radiation (λ = 1.54184 Å). SADABS absorption correction was applied to the data for **2** and **14** [2]. For **5**, analytical numeric absorption correction using

a multifaceted crystal model was performed with the CrysAlisPro program package [3,4]. The structures were solved by direct methods with the SIR97 [5] and SHELXS-97 [6] programs, and full-matrix, least-squares refinements on F^2 were performed, using the SHELXL-97 [6] program. Molecular structure figures were drawn with ORTEP3 for Windows [7]. In compound **14**, the water molecule in the lattice on the two-fold axis showed a population parameter of 0.3. The full occupation in this position is 0.5.

References

1. Kiss, L.; Forró, E.; Fülöp, F. *Tetrahedron* **2012,** *68,* 4438. doi:10.1016/j.tet.2011.12.065
2. *SADABS*, Sheldrick, G. M.; University of Göttingen: Germany, 2008.
3. Clark, R. C.; Reid, J. S. *Acta Crystallogr., Sect. A* **1995,** *51,* 887–897. doi:10.1107/S0108767395007367
4. CrysAlis PRO, 2012, Agilent Technologies, Yarnton, England.
5. Altomare, A.; Burla, M. C.; Camalli, M.; Cascarano, G. L.; Giacovazzo, C.; Guagliardi, A.; Moliterni, A. G. G.; Polidori, G.; Spagna, R. *J. Appl. Crystallogr.* **1999,** *32,* 115. doi:10.1107/S0021889898007717
6. Sheldrick, G. M. *Acta Crystallogr., Sect. A* **2008,** *64,* 112. doi:10.1107/S0108767307043930
7. Farrugia, L. J. *J. Appl. Crystallogr.* **1999,** *32,* 837. doi:10.1107/S0021889899006020
